# A case of delayed dyspnea after corrective posterior fusion of the middle and lower cervical spine for dropped head syndrome

**DOI:** 10.1093/jscr/rjae047

**Published:** 2024-02-16

**Authors:** Shinsuke Sato, Yusuke Nakao, Shingo Kumaki, Shigeo Sano

**Affiliations:** Department of Orthopaedic Surgery, Sanraku Hospital, 2-5, Kandasurugadai, Chiyoda-ku, Tokyo 101-8326, Japan; Department of Orthopaedic Surgery, Sanraku Hospital, 2-5, Kandasurugadai, Chiyoda-ku, Tokyo 101-8326, Japan; Department of Orthopaedic Surgery, Sanraku Hospital, 2-5, Kandasurugadai, Chiyoda-ku, Tokyo 101-8326, Japan; Department of Orthopaedic Surgery, Sanraku Hospital, 2-5, Kandasurugadai, Chiyoda-ku, Tokyo 101-8326, Japan

**Keywords:** dyspnea, kyphosis, kyphoscoliosis, lordosis, posterior fixation, spinal fusion

## Abstract

Dyspnea has been reported to occur following posterior occipitocervical fusion. However, there are no documented cases of dyspnea following posterior fixation of the middle and lower cervical spine without posterior occipitocervical fusion. An 80-year-old woman underwent corrective fusion from T4 to the ilium for kyphoscoliosis. Sixteen months later, the patient developed cervical kyphosis (dropped head syndrome) with proximal junctional kyphosis, leading to a pedicle subtraction osteotomy at T4 and an extended fixation to C2. On the sixth postoperative day, the patient experienced respiratory arrest, prompting a reoperation to reduce cervical lordosis, ultimately resolving the respiratory dysfunction. Excessive correction of cervical kyphosis should be avoided to prevent the occurrence of postoperative dyspnea, even in cases where posterior occipitocervical fusion has not been performed.

## Introduction

Dyspnea following posterior occipitocervical fusion (OCF) is often attributed to upper airway obstruction resulting from a reduction in the pharyngeal space [1]. Dyspnea may rarely occur following OCF, especially after incorrect alignment. We present a case of delayed-onset dyspnea following posterior fusion of the middle and lower cervical spine without OCF and discuss the potential causes.

## Case report

**Figure 1:**
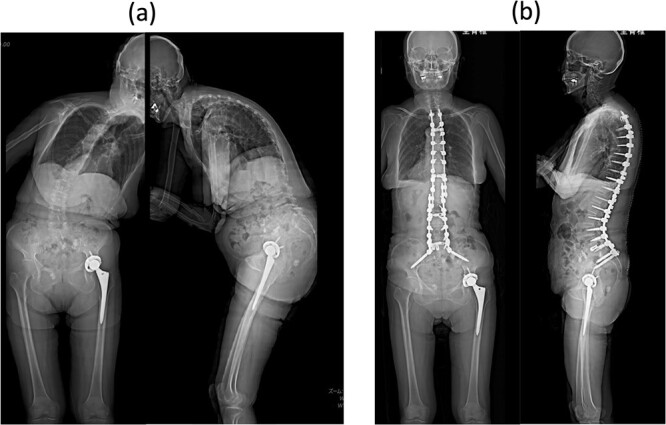
Standing whole-spine anteroposterior and lateral radiographs (a) preoperative and (b) after the first surgery.

An 80-year-old woman presented to our department complaining of back and leg pains, as well as postural imbalance. She could stand continuously for 10 min and experienced intermittent claudication after walking 300 m. She was diagnosed with kyphoscoliosis and lumbar spinal canal stenosis and subsequently underwent corrective spinal fusion surgery from T4 to the ilium (Fig. 1).

**Figure 2:**
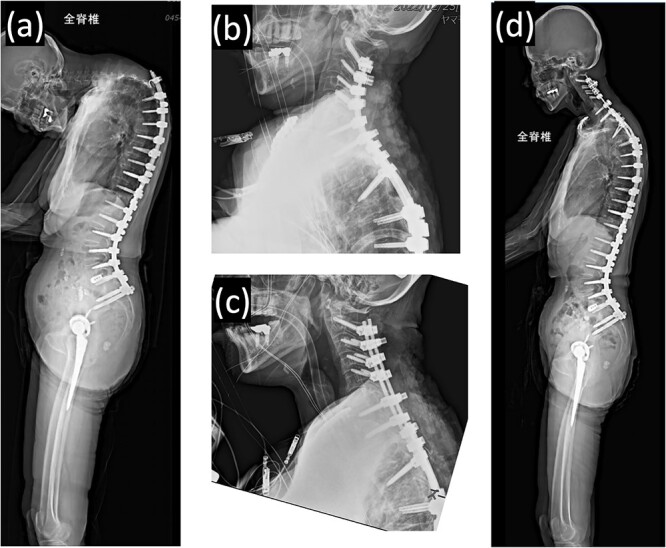
(a) Standing whole-spine radiographs 16 months after the first surgery, showing features of dropped head syndrome associated with proximal junctional failure; lateral radiographs immediately after (b) the second and (c) third surgeries; (d) standing whole-spine radiographs 16 months after the third surgery.

Proximal junctional kyphosis gradually developed after the initial surgery, eventually causing difficulty in maintaining a horizontal gaze 16 months post-surgery due to dropped head syndrome (Fig. 2a). A second corrective cervical spine surgery was performed 17 months post-surgery, involving extended fusion to C2 and pedicle subtraction osteotomy of T4 (Fig. 2b). The patient was extubated in the operating room immediately after the surgery and returned to the ward.

**Figure 3:**
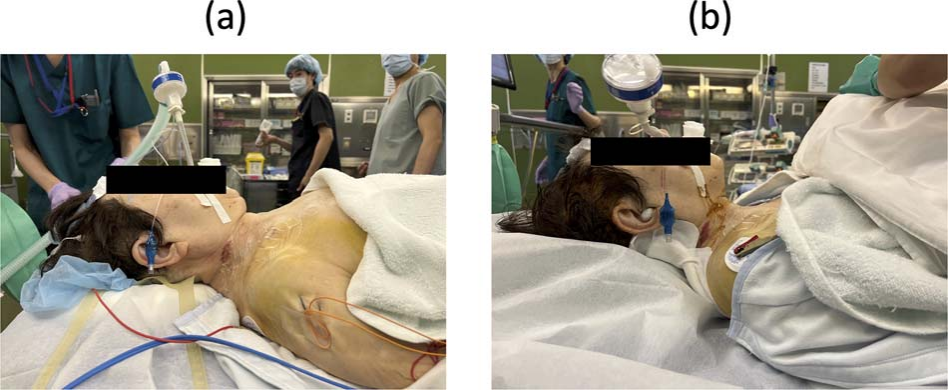
Lateral view of neck posture (a) before and (b) after the third surgery.

On postoperative Day 1, she resumed eating and drinking, but with delayed swallowing time. The wound drain was removed on postoperative Day 2, and walking exercises were initiated. Though no specific issues were noted, she exhibited signs of delirium, unclear responses, and occasional snoring-like breathing. However, on postoperative Day 6, a nurse found her bedridden, with a pale face and eyes open, drooling and snoring. The patient’s oxygen saturation was 55%; when the bed was flattened and the airway was suctioned, consciousness was restored and oxygenation improved in ~20 s. The diagnosis was obstructive dyspnea, supported by the persistence of snoring after resuming breathing. Neurological symptoms had not worsened, and intracranial lesions or cardiovascular events were deemed unlikely based on computed tomography (CT) scans, electrocardiograms, and blood test results. The rapid increase in the C2–7 angle from −15° before the second operation to 32° postoperatively led to the conclusion that a decrease in the O-C2 angle was associated with occlusive dyspnea. Emergency surgery was performed to re-fixate the cervical spine, reducing kyphosis (Fig. 2c). Postoperatively, the patient was managed on a ventilator and extubated on the second postoperative day. Sixteen months later, she experienced no further dyspnea (Fig. 2d and 3b).

## Discussion

Several reports have described dyspnea and dysphagia following posterior OCF, associated with a reduced O-C2 angle [1]. There have been no previous reports of dyspnea following middle and lower cervical fixation without OCF, although dysphagia has been reported [2]. Here, we explore potential causes of dyspnea in our case.

Dyspnea following OCF is attributed to the narrowing of the pharyngeal airway due to fixation in the flexed position [1]. The pharyngeal airway diameter correlates with the change in the O-C2 angle, not the C2–7 angle [2, 3]. Fixation of O-C2 at an angle reduced by >10° is a risk factor for breathing and swallowing problems [4]. However, the C2–7 angle cannot be arbitrarily fixed as it is negatively correlated with the O-C2 angle [5, 6], making finding an ideal indicator for correcting cervical kyphosis challenging.

**Figure 4:**
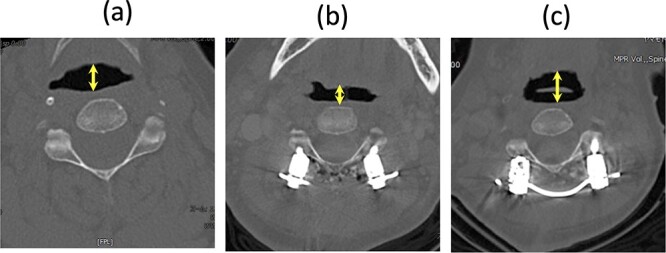
CT axial image at the C2 level in a supine position; (a) before the second surgery, (b) after the second surgery, and (c) after the third surgery; the arrow shows the pharyngeal diameter.

In our case, the C2–7 angle increased from −15° before the second surgery to 32° after the surgery. Considering the relationship between the O-C2 and C2–7 angles in healthy individuals, we expected the corrected O-C2 angle to be ~0°. The first potential cause of dyspnea was a significant reduction in this angle by 19° after the second surgery. Although the O-C2 angle was not fixed, if the patient’s head were retracted, for example, due to sliding down the bed with a higher pillow, the reduced O-C2 angle might have led to obstructive dyspnea, exacerbated by the patient’s delirium. The pharyngeal diameter at the C2 level on the supine CT scan decreased immediately after dyspnea and resuscitation, widening again after the final surgery (Fig. 4). A comparison of the lateral radiograph before and after the second surgery showed that the C2–7 angle increased significantly from −15° to 32°. However, standing radiographs were not taken after the second surgery and the O-C2 angle was not measured. The negative correlation between the O-C2 and C2–7 angles predicts that the O-C2 angle tends to be smaller [5, 6], even in normal standing and sitting positions (Table 1).

**Table 1:** Changes in spinal parameter.

	Preoperative	After first surgery	Before second surgery	After second surgery	After third surgery
O-C2 angle (°)	8	7	19	-	19
C2–7 angle (°)	14	41	−15	32	5
T1 slope (°)	47	36	70	-	44
Thoracic Kyhosis (°)	74	60	97	-	58
Lumbar Lordosis (°)	20	62	62	62	62
Pelvic Tilt (°)	24	12	24	-	20
Pelvic Incidence (°)	60	60	60	60	60
C2–7 SVA (mm)	12	14	90	-	45
C7-SVA (mm)	320	7	−8	-	14

No standing X-rays were taken after second surgery.

SVA, Sagittal Vertical Axis.

The second potential cause of dyspnea was related to pelvic fixation. If the lower instrumented vertebra (LIV) is not pelvic, bending the distal part more than the LIV can increase the O-C2 angle, possibly aiding airway security. In this case, fixation from C2 to the ilium left the only mobile segment as O-C2, making compensation below the hip joint difficult. Pelvic fixation was initially performed in the present case, suggesting the need for careful attention to correcting the cervical spine in dropped head syndrome after adult spinal deformity (ASD) surgery.

Alignment indices for correcting dropped head syndrome include C2–7 sagittal vertical axis of <40 mm, T1 slope of <40°, and T1 slope-CL of <20° [7]. However, these parameters are used in the standing position; no clear intraoperative indices exist. Kaneyama *et al*. [8] proposed the S-line as a reference, which can be measured intraoperatively during OCF and can indicate a risk of dysphagia when it is posterior to the apex of the cervical lordosis. Mandibular retraction is a risk factor for postoperative dysphagia [9]. Soft tissue retraction anterior to the cervical spine is a dysphagia risk factor when the C2–7 angle increases by >5° [10]. In our case, the occipitocervical spine was not fixed, so the S-line is a helpful but not accurate indicator. When the occipitocervical spine is not fixed, the O-C2 angle changes depending on the patient’s posture. The sniffing position is advantageous for opening the airway [11]. Although it is inadequate for correcting dropped head syndrome, safety should be a priority rather than an obsession with obtaining cervical kyphosis.

In conclusion, we recommend avoiding overcorrection of the middle and lower cervical spine in cases of dropped head syndrome, even when O-C2 is not fixed.

## References

[1] Yoshida M , Neo M, Fujibayashi S, Nakamura T. Upper-airway obstruction after short posterior occipitocervical fusion in a flexed position. Spine (Phila Pa 1976) 2007;32:E267–70. 17426623 10.1097/01.brs.0000259977.69726.6f

[2] Ishikawa Y , Miyakoshi N, Hongo M, et al. Recurrent dysphagia after lower posterior cervical fusion. Surg Neurol Int 2020;11:114. 32494389 10.25259/SNI_194_2020 PMC7265380

[3] Izeki M , Neo M, Takemoto M, et al. The O-C2 angle established at occipito-cervical fusion dictates the patient’s destiny in terms of postoperative dyspnea and/or dysphagia. Eur Spine J 2014;23:328–36. 23982903 10.1007/s00586-013-2963-6 PMC3906459

[4] Miyata M , Neo M, Fujibayashi S, et al. O-C2 angle as a predictor of dyspnea and/or dysphagia after occipitocervical fusion. Spine (Phila Pa 1976) 2009;34:184–8. 19139669 10.1097/BRS.0b013e31818ff64e

[5] Lee SH , Kim KT, Seo EM, et al. The influence of thoracic inlet alignment on the craniocervical sagittal balance in asymptomatic adults. J Spinal Disord Tech 2012;25:E41–7. 22037167 10.1097/BSD.0b013e3182396301

[6] Inoue T , Ito K, Ando K, et al. Age-related changes in upper and lower cervical alignment and range of motion: normative data of 600 asymptomatic individuals. Eur Spine J 2020;29:2378–83. 32720125 10.1007/s00586-020-06547-9

[7] Ling FP , Chevillotte T, Leglise A, et al. Which parameters are relevant in sagittal balance analysis of the cervical spine? A literature review Eur Spine J 2018;27:8–15. 29332239 10.1007/s00586-018-5462-y

[8] Kaneyama S , Sumi M, Takabatake M, et al. The prediction and prevention of dysphagia after Occipitospinal fusion by use of the S-line (swallowing line). Spine 2017;42:718–25. 27779604 10.1097/BRS.0000000000001963

[9] Izeki M , Neo M, Ito H, et al. Reduction of atlantoaxial subluxation causes airway stenosis. Spine 2013;38:E513–20. 23392412 10.1097/BRS.0b013e31828b26df

[10] Tian W , Yu J. The role of C2-C7 and O-C2 angle in the development of dysphagia after cervical spine surgery. Dysphagia 2013;28:131–8. 22918711 10.1007/s00455-012-9421-1

[11] Isono S , Tanaka A, Ishikawa T, et al. Sniffing position improves pharyngeal airway patency in anesthetized patients with obstructive sleep apnea. Anesthesiology 2005;103:489–94. 16129972 10.1097/00000542-200509000-00010

